# Oral Erythema Multiforme: A Case Report and Review of Diagnostic, Therapeutic and Prognostic Challenges

**DOI:** 10.7759/cureus.66749

**Published:** 2024-08-13

**Authors:** Deivanayagi M, Narmadha Chandran, Elamparithi B, Sakthi S, Thennarasu A R

**Affiliations:** 1 Oral Medicine and Radiology, Adhiparasakthi Dental College and Hospital, Melmaruvathur, IND; 2 Oral and Maxillofacial Surgery, Adhiparasakthi Dental College and Hospital, Melmaruvathur, IND

**Keywords:** autoimmune like, corticosteroids, oral mucosal disease, case report, erythema multiforme

## Abstract

Erythema multiforme (EM) presents a distinct challenge in both diagnosis and management, particularly when its manifestations extend to the oral cavity. Classified into "minor" and "major" forms based on clinical presentation, EM defies gender bias and tends to affect individuals across different age groups. The complexity arises from its varied symptoms within the oral cavity, where it commonly manifests as painful, red erosive plaques known as target lesions, primarily observed on the lips and oral mucosa. These lesions may arise independently or be linked to underlying systemic or infectious conditions, complicating the diagnostic process. Here, we present a case study of a 55-year-old female patient grappling with EM, underscoring the importance of meticulous clinical examination, thorough investigations, tailored treatment strategies, and subsequent outcomes.

## Introduction

Erythema multiforme (EM) is a rare acute condition affecting both the skin and mucous membranes, triggered by an immune response. The presence of cytotoxic T lymphocytes in the epidermis initiates cell death in keratinocytes, resulting in satellite cell necrosis and the distinctive appearance of EM lesions [1]. It is characterized by the presence of lesions that are often accompanied by erosions or blisters affecting the oral, genital, and/or ocular mucosa. When these mucosal lesions are severe, it is referred to as EM major, and may also be associated with systemic symptoms such as fever and joint pain [2]. In contrast, EM with mild or no mucosal involvement and no systemic symptoms is referred to as EM minor or simply EM without major mucosal disease [3]. Other than this, toxic epidermal necrolysis (TEN) and Steven-Johnson syndrome (SJS) are the other variants of EM. This immune-driven disorder is marked by the emergence of bull's-eye-shaped lesions on the skin, EM and related conditions fall into a category involving inflammation of both mucous membranes and skin, resulting in varying degrees of blisters and ulcers. In severe cases, these disorders can manifest systemic symptoms, posing a threat to overall health [3]. In the oral cavity, EM frequently affects the lips followed by the buccal mucosa and tongue. Ocular and genital involvement can also be associated with the mucosal type of EM. TEN and SJS are different systemic conditions that share some clinical similarities with EM. These disorders share similar mucosal lesions and differ from cutaneous lesions occurrence [4].

This paper details a case of EM affecting a female patient, characterized by severe oral ulcerations, which presents a prognostic challenge when managed conservatively. Additionally, we aim to explore the existing diagnostic, therapeutic, and prognostic hurdles associated with EM through a concise literature review.

## Case presentation

**Figure 1:**
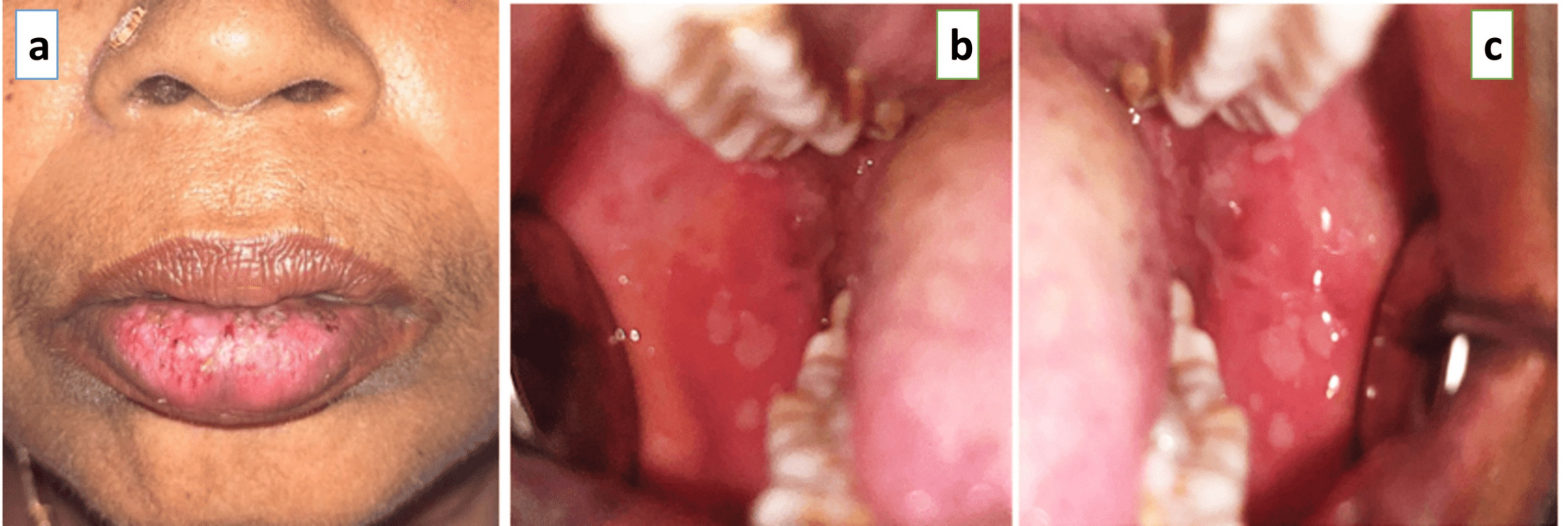
A: Necrotic encrustations in the lower lip; B and C: Ulcerations involving the right and left buccal mucosa

A 55-year-old female patient reported to the Department of Oral Medicine and Radiology with a chief complaint of painful ulcers in the mouth for six months associated with a burning sensation. The patient gave a history of medication using topical triamcinolone acetonide 0.1% for the same complaint after a previous dental consultation. The patient also had a history of occurrence of multiple ulcers in the lower lip associated with worsened symptoms for the past three months. Her medical history was non-contributory. The patient's general status revealed that the patient is well-oriented and moderately nourished. On intra-oral soft tissue examination, there was evidence of multiple ulcers coalescence to represent a single lesion involving the lower lip. The surface of the lesion has necrotic encrustations with active bleeding spots (Figure 1A). There was evidence of multiple ulcers of size 3x4 mm involving the right and left buccal mucosa with the surface appearing yellowish and erythematous halo (Figures 1B, 1C). On palpation, the ulcerated areas were tender. Based on the clinical history and examination, EM was considered a provisional diagnosis. The herpetic ulcer was considered a differential diagnosis. 

**Figure 2:**
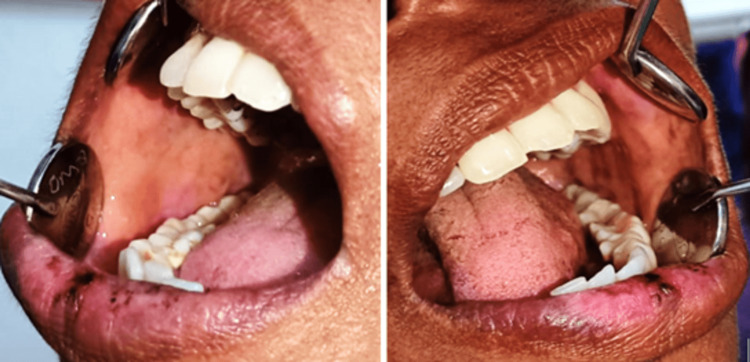
Images depicting partial remission of lip lesions and complete remission of lesions in the right and left buccal mucosa

The patient initially received treatment with 5 mg of prednisolone for one week and topical triamcinolone acetonide 0.1%. Investigations were conducted to rule out herpes simplex virus (HSV) association, and routine blood parameters were assessed, revealing non-reactive serum IgG and IgM HSV and normal blood investigations. After one week, all lesions persisted, prompting a revised treatment of acyclovir 400 mg and 5 mg of prednisolone twice daily for two weeks. In the third review, partial remission of lip lesions and complete remission of buccal mucosa lesions were observed (Figure 2).

**Figure 3:**
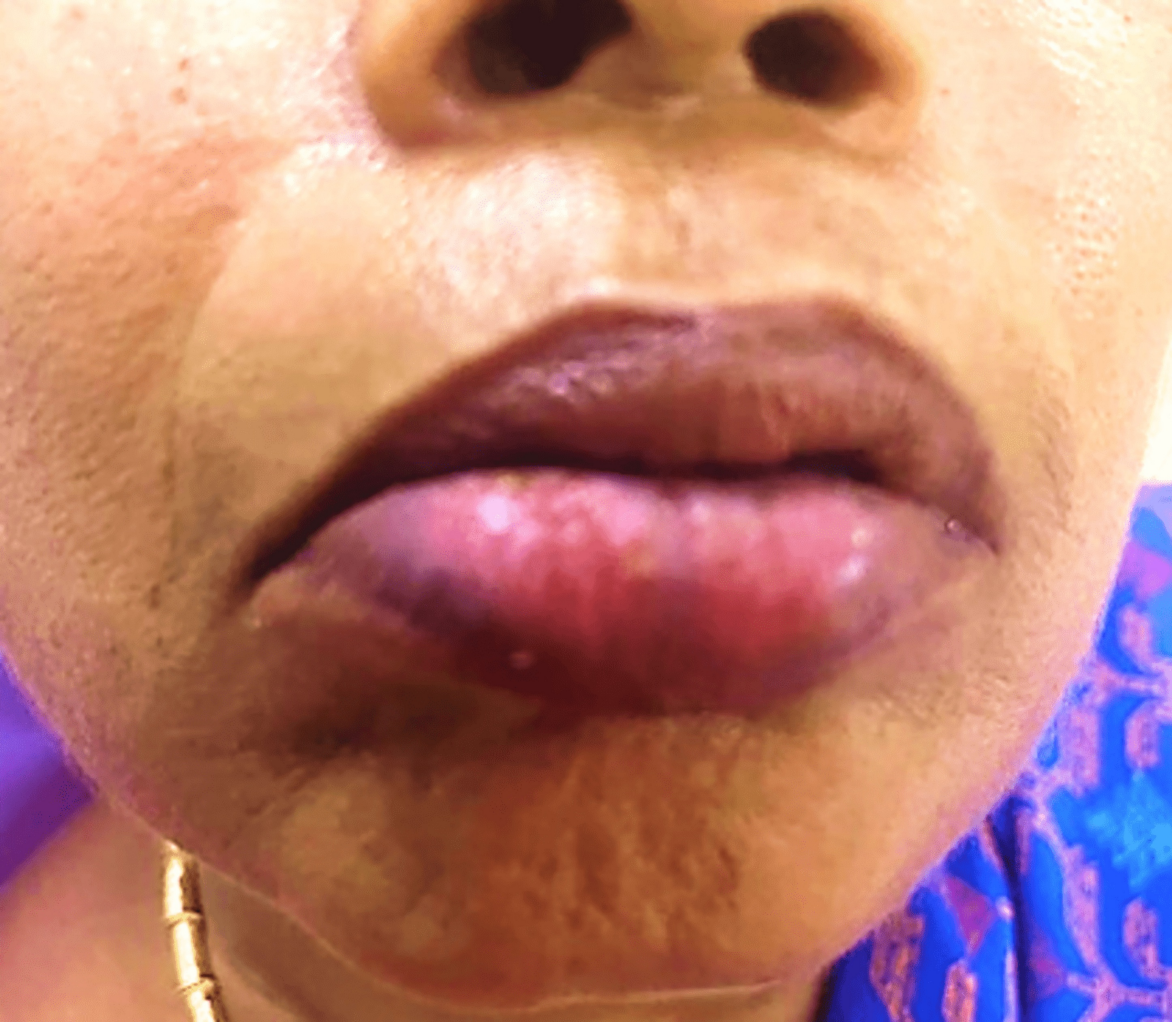
Image showing complete remission of the lesion in the lower lip

Consequently, the prednisolone dose was increased to 10 mg twice daily. In the follow-up review, partial remission of lip lesions continued with no recurrence of buccal mucosa lesions. The patient was then managed with low-dose prednisolone (5 mg twice daily) and levamisole 150 mg once daily for one week. After one week, complete regression of lesions on both the lips and buccal mucosa was noted (Figure 3).

**Figure 4:**
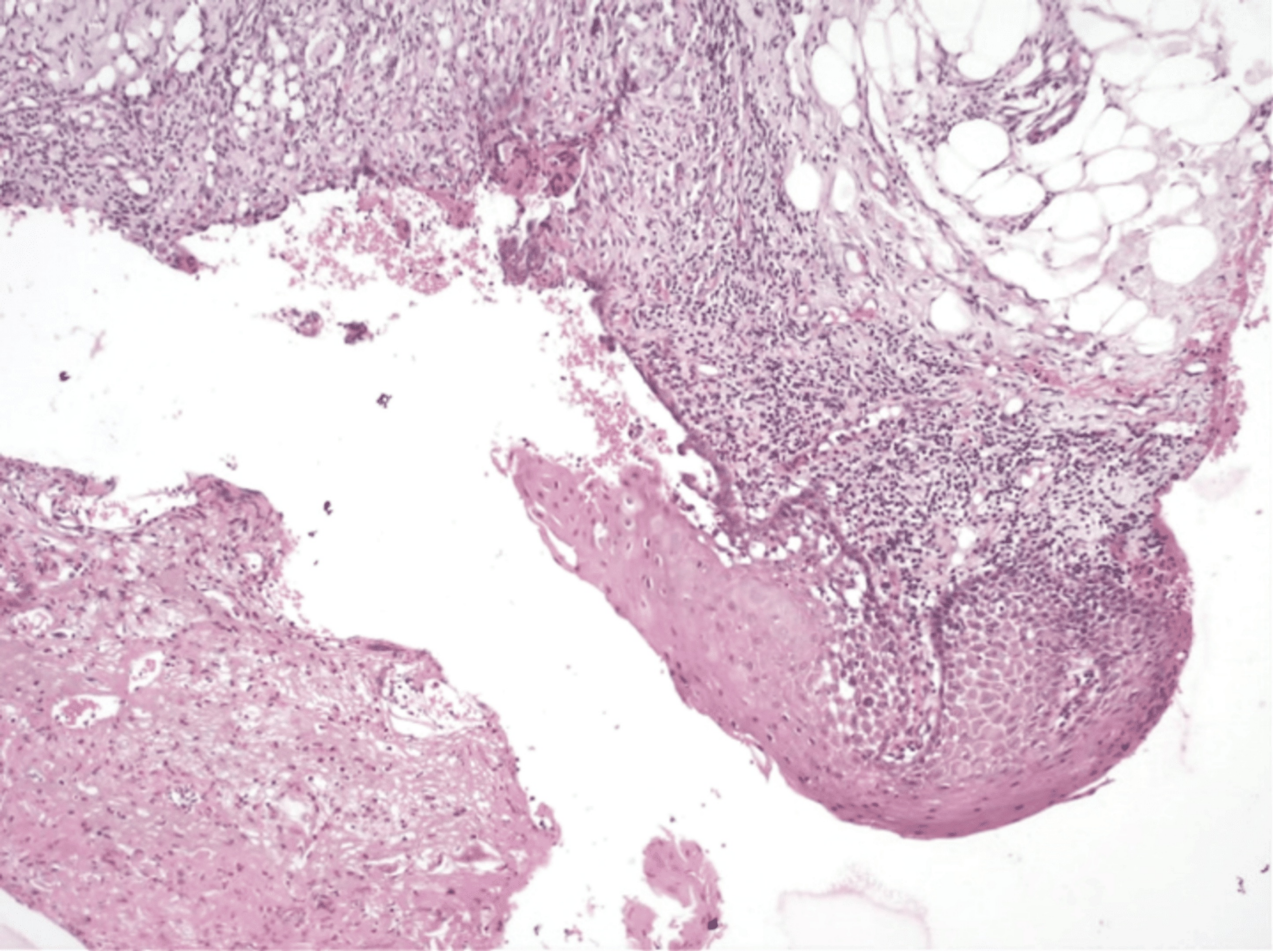
Photomicrograph of the 40X - H and E section showing non-keratinized stratified squamous epithelium ulcerated with separation of spinous cells and degeneration, showing a split between the epithelium and connective tissue.

In the meantime, the patient underwent punch biopsy under standard aseptic protocols. The histopathological features (Figure 4) confirm the diagnosis of EM which showed non-keratinized stratified squamous epithelium ulcerated with separation of spinous cells and degeneration, showing a split between the epithelium and connective tissue. The basal cells have undergone degeneration and the superficial layers show spongiosis with inter and intra-cellular edema. There were extravasated blood cells and inflammatory infiltrate.

## Discussion

This case posed a tremendous prognostic challenge which involved the administration of steroids, antiviral drugs, and levamisole (anthelminthic) drug. The approach to treating EM can vary based on the severity of symptoms, the underlying cause, and whether the condition is acute or chronic [5]. Before initiating symptomatic treatment, it's crucial to identify the underlying cause or triggering medication/infection in EM cases. In such instances, the cessation of the medication or appropriate treatment of the infection is prioritized. This is because different factors can influence the presentation and progression of EM, and therefore, tailored treatment plans may be necessary [2].

Diagnostic challenges

Diagnosing immune-mediated oral mucosal lesions poses inherent challenges. While biopsy remains the gold standard for definitive diagnosis, it's imperative to thoroughly investigate and rule out underlying systemic diseases. Earlier, EM was classified into four distinct types: EM minor, EM major, SJS, and toxic epidermal necrolysis (TEN). However, as per updated literature, SJS and TEN are now recognized as separate entities rather than variants of EM since they have different mucocutaneous clinical presentations [4]. An effective diagnostic approach involves excluding potential medical histories where applicable. Viral aetiologies, notably HSV, necessitate comprehensive history-taking and serological testing for IgG and IgM antibodies. HSV-induced EM often presents with prodromal symptoms and is among the most common EM variants [6,7]. Notably, EM lacks association with specific immunologic patterns or serologic abnormalities commonly observed in autoimmune diseases [8]. A multidisciplinary approach, involving oral physicians, dermatologists, and pathologists, is essential for formulating a definitive diagnosis and guiding appropriate management strategies.

Therapeutic challenges

Managing EM presents challenges as it necessitates addressing the underlying cause. Various aetiologies have been documented, including viral-induced [e.g., HSV], bacterial-induced (e.g., Mycoplasma pneumoniae), and drug-induced (e.g., NSAIDs, antibiotics, immunoglobulins, antiepileptics, sulphonamides) [2,9,10]. Recent studies have suggested a potential association between EM and novel coronavirus infection [11]. Additionally, less common etiological factors include candidiasis, hepatitis C infection, food preservatives, puberty, menstruation, and menopausal status [12,13]. The treatment strategies for EM are based on the occurrence of the lesion as an acute or recurring lesion.

HSV-induced EM

The current modality of treatment is antiviral drugs acyclovir 800 mg/day, valacyclovir 500 mg twice daily, or famciclovir, 250 mg, twice daily for seven days [14].

EM due to drugs/allergic condition

Oral corticosteroids represent the primary treatment modality for EM, with methylprednisolone typically initiated at a minimum dosage of 20 mg/day and titrated up to 60 mg/day as necessary. The dosage should be gradually tapered over 2-4 weeks [15]. In cases where patients do not respond to corticosteroids, alternative medications such as dapsone, azathioprine, and levamisole may be considered. Additionally, antihistamines like cimetidine (800 mg/day) and cetirizine (10 mg/day) are recommended as adjunctive therapy to alleviate symptoms [14].

Levamisole can be administered at a dosage ranging from 50 to 150 mg as a single daily dose for the treatment of EM [16]. Similarly, dapsone can be prescribed at dosages ranging from 50 to 100 mg per day for the management of EM [17].

EM due to systemic diseases

Effective management of this disease necessitates addressing underlying systemic diseases, particularly in cases where conditions like inflammatory bowel disease and malignancies such as leukemia contribute to recurrent or persistent EM [18]. Immunomodulators play a crucial role in such scenarios. Medications such as sdalimumab, cyclosporine, mycophenolate mofetil, thalidomide, and immunoglobulins are recommended for managing recalcitrant EM lesions [3]. Employing these drugs requires a multidisciplinary approach involving oral physicians, immunologists, and medical oncologists to ensure comprehensive care and treatment optimization.

Severe and recrudescent EM

Severe cases of EM accompanied by oral mucosal involvement and significant fluid loss necessitate systemic intervention. This includes administration of intravenous fluids and fluid replacement therapy to address dehydration. Additionally, intravenous immunomodulators may be required to manage the immune response [2]. In recrudescent cases, a two-week course of antiviral therapy is essential, followed by steroid therapy with a carefully planned tapering schedule to prevent rebound effects and ensure optimal management of symptoms [14,15,18].

Prognostic challenges

The prognosis of EM hinges on effectively addressing the underlying cause. Acute EM cases typically resolve spontaneously, with treatment strategies primarily focusing on corticosteroids and antihistamines. However, the case discussed in this paper posed significant prognostic challenges. The treatment approach commenced with low-dose steroid therapy, followed by antiviral therapy, and moderate-dose steroid therapy, and concluded with levamisole administration. Proper follow-up and avoidance of exacerbating factors are crucial for determining prognostic outcomes in EM cases. The recurrence rate of EM is less than 5%, with most recurrences associated with HSV infections.

The treatment options have received limited discussion in the literature, with some case series and cohort studies exploring drugs such as acyclovir, levamisole, dapsone, thalidomide, and corticosteroids [14-16,19,20].

## Conclusions

The treatment options for this autoimmune disease are limited and controversial. Additionally, immunomodulators like rituximab and apremilast have been investigated for EM treatment. However, the studies supporting these drugs lack confirmation from randomized controlled trials, highlighting the need for a more evidence-based approach to address therapeutic and prognostic challenges in EM management. Moreover, a multidisciplinary approach involving oral physicians, dermatologists, and immunologists remains crucial for comprehensive EM management.
